# Tailoring convolutional neural networks for custom botanical data

**DOI:** 10.1002/aps3.11620

**Published:** 2024-10-21

**Authors:** Jamie R. Sykes, Katherine J. Denby, Daniel W. Franks

**Affiliations:** ^1^ Department of Computer Science University of York Deramore Lane York YO10 5GH Yorkshire United Kingdom; ^2^ Centre for Novel Agricultural Products, Department of Biology University of York Wentworth Way York YO10 5DD Yorkshire United Kingdom; ^3^ Department of Biology University of York Wentworth Way York YO10 5DD Yorkshire United Kingdom

**Keywords:** disease detection, machine learning, plant pathology, spectroscopy

## Abstract

**Premise:**

Automated disease, weed, and crop classification with computer vision will be invaluable in the future of agriculture. However, existing model architectures like ResNet, EfficientNet, and ConvNeXt often underperform on smaller, specialised datasets typical of such projects.

**Methods:**

We address this gap with informed data collection and the development of a new convolutional neural network architecture, PhytNet. Utilising a novel dataset of infrared cocoa tree images, we demonstrate PhytNet's development and compare its performance with existing architectures. Data collection was informed by spectroscopy data, which provided useful insights into the spectral characteristics of cocoa trees. Cocoa was chosen as a focal species due to the diverse pathology of its diseases, which pose significant challenges for detection.

**Results:**

ResNet18 showed some signs of overfitting, while EfficientNet variants showed distinct signs of overfitting. By contrast, PhytNet displayed excellent attention to relevant features, almost no overfitting, and an exceptionally low computation cost of 1.19 GFLOPS.

**Conclusions:**

We show that PhytNet is a promising candidate for rapid disease or plant classification and for precise localisation of disease symptoms for autonomous systems. We also show that the most informative light spectra for detecting cocoa disease are outside the visible spectrum and that efforts to detect disease in cocoa should be focused on local symptoms, rather than the systemic effects of disease.

## BACKGROUND

Computer vision projects in areas like plant pathology and agronomy often have limited data for training and are thus constrained in the choice of model architecture. ResNet (He et al., 2016), EfficientNet (Tan and Le, 2020), and ConvNeXt (Liu et al., 2022) variants are among the best current neural networks available for such smaller datasets (Sykes et al., 2024). However, these models were developed for general image recognition tasks and are honed to perform well on huge benchmark datasets such as ImageNet, which has 1.4 million images and 1000 classes (Woo et al., 2023). In recent years of model development, the focus has moved away from convolutional neural networks (CNNs) towards the larger transformer and CNN‐transformer hybrid models such as Detection Transformer (DETR) (Zhu et al., 2021). While this trend has yielded numerous highly performant models, it leads to problems of scale for many real‐world applications. The performance of transformers like the Vision Transformer (ViT) and larger CNNs like ConvNeXt scales exceptionally well with large datasets. However, training these models and curating the requisite massive training datasets is prohibitively expensive. Additionally, the increased runtime cost of these models necessitates fast internet connections and/or expensive hardware (Zhang et al., 2022), further prohibiting their use. The authors of ViT acknowledge such issues for models like Swin Transformer (Liu et al., 2021) and Vision Mixture of Experts (V‐MoE; Riquelme et al., 2021), which have three and 14.7 billion parameters, respectively. However, their solutions, mini‐ViT (Zhang et al., 2022) and tiny‐ViT (Wu et al., 2022) still have tens of millions of parameters. As well as choosing a model variant like ConvNeXt Tiny or ConvNeXt Large, it is relatively simple to adjust the size of ConvNeXt and EfficientNet modes yourself by simply adjusting the “block_settings” parameter in ConvNeXt or the “width_mult” and “depth_mult” parameters of EfficientNet. However, in doing so, you forfeit the benefit of the pre‐trained ImageNet weights, which will no longer fit the newly scaled model, so these large models must be trained from scratch. This will produce poor results with a modest‐sized dataset. We found that adjusting the scale parameters of ConvNeXt had severe negative effects on performance.

### Fitting architectures to datasets

In projects with small training datasets, it is crucial to match the neural network's size to the size of the dataset. This will help prevent overfitting and promote effective generalisation. Without this, the model may memorise the training data, including its noise and outliers, rather than learning genuine patterns. This will result in poor performance on new data and give a misleading sense of model accuracy based on performance metrics observed during training.

We took inspiration from the design of ResNet, ConvNeXt, and EfficientNet to develop a state‐of‐the‐art model, PhytNet, and an accompanying hyperparameter search. The aim was to produce a CNN that would perform well on realistically sized datasets and have minimal computation cost at runtime. CNNs have several intrinsic benefits over transformers that, for image classification, allow CNNs to outperform transformers while requiring fewer parameters and far less data for training (Liu et al., 2022).

Many of the recent performance gains in image classification have come from novel training procedures involving semi‐supervised and unsupervised pre‐training (Woo et al., 2023; Sykes et al., 2024). While we intend to apply such techniques to the pre‐training of PhytNet with a custom plant dataset, that work is beyond the scope of this study and will be presented in a separate future study. Here we aim first to define a small, high‐quality dataset and use it to guide the development of a new model architecture. With PhytNet, we bring both the best‐established features and the latest advancements to small model design. In doing so, we also report observations regarding the localisation of machine‐visible disease symptoms in cocoa trees and identify the most informative parts of the ultraviolet to infrared electromagnetic spectrum for cocoa disease detection. These results will be informative for the development of future training datasets and models as we continue to search for informative signals of disease. Lastly, we apply cross‐validation and gradient‐based class activation maps (Grad‐CAM) (Selvaraju et al., 2017) to compare and contrast PhytNet against ResNet18, EfficientNet‐b0, EfficientNet‐V2s, and ConvNeXt Tiny.

### PhytNet development

To develop PhytNet and demonstrate the practical means of its development, we use the real‐world challenge of classifying images of disease in cocoa trees, *Theobroma cacao* L. Cocoa is highly vulnerable to fungal and oomycete diseases due to the humid conditions in which it grows. Most disease control in cocoa involves costly manual search and phytosanitation (Meinhardt et al., 2008). Because most cocoa farmers live below the poverty line, they cannot easily afford such expenses or those of chemical pesticides (Boeckx et al., 2020). Each year, about 38% of the global cocoa crop is lost to three main diseases: black pod rot, 23% (BPR; *Phytophthora palmivora*); witches' broom disease, 13% (WBD; *Moniliophthora perniciosa*); and frosty pod rot, 2% (FPR; *Moniliophthora roreri*) (Marelli et al., 2019). These three diseases, which impose a significant burden on cocoa farmers and, indirectly, on the natural environment (Malhi et al., 2008; Kuok Ho and Yap, 2020), will be of central concern in this study. The most common symptoms of these three diseases are as follows—BPR: black or brown lesions on the pod exterior, pod mummification, and premature dropping of fruit; WBD: malformed skin and abnormal, although characteristic, fruit shape, black or brown lesions on the fruits, and malformation of stems, leaves, and flowers; FPR: abnormal, although characteristic, fruit shape, discolouration of pods, extensive growth of white mycelium, black or brown lesions on the pod, liquefaction of pod contents, and mummification of pods. Cocoa is a prime use case for automated disease detection as its diversity of diseases with overlapping and cryptic symptoms pose a significant challenge for human and machine disease classifiers. This makes it an ideal challenge by which to guide the development of a phytology‐focused classification model.

## METHODS

### Spectroscopy

We used a MultispeQ v2.0 (PhotosynQ, East Lansing, Michigan, USA) (Kuhlgert et al., 2016) to measure photosystem II quantum yield (Phi2) and non‐photochemical quenching (NPQt) in cocoa trees with different disease states. Both Phi2 and NPQt have been shown to have significant negative and positive correlations with disease index, respectively (Kuhlgert et al., 2016). These measurements were taken to assess if non‐visible signals of disease could be detected in the foliage of cocoa trees. Readings were taken from (1) healthy leaves on trees with no signs of disease nearby (*n* = 9); (2) leaves clearly infected with WBD (*n* = 5); and (3) the nearest leaf to pods or leaf tissue infected with BPR (*n* = 10), FPR (*n* = 5), or WBD (*n* = 5). Measurements were taken across four sites northeast to southeast of Guayaquil, Ecuador.

To measure the reflectance spectrum of diseased and healthy cocoa pods, we used a field spectrometer produced by tec5 (Steinbach, Germany). This spectrometer had a receptive range of 310–1100 nm. The spectroscopy data were gathered on four farms located east of Guayaquil, Ecuador, near the foothills of the Andes Mountains. Readings were taken following the manufacturer's instructions. We trained a random forest classifier to predict disease state from the reflectance data to estimate the relative importance of different spectral bands. Hyperparameter values for the random forest were chosen using a grid search with fivefold cross‐validation. The test accuracy of this classifier was 78%. We considered only healthy cocoa pods (*n* = 47) and pods with clear symptoms of BPR (*n* = 40) or FPR (*n* = 24). No WBD was present in the cocoa pods at these plantations due to the time of year.

### Image dataset collection

We collected two datasets of infrared (*n* = 240) and red, green, blue (RGB) (*n* = 240) images taken concurrently with the same Olympus OMD EM‐5 II camera (Tokyo, Japan). While the infrared data were collected primarily for model development, the additional RGB data allowed us to compare two ResNet18 models for disease classification—one trained on infrared images and the other trained on RGB images. Images were of healthy cocoa trees bearing pods as well as trees bearing pods with BPR, FPR, and WBD in equal numbers. Diseased pods showed clearly visible and easily diagnosable symptoms in the early to middle stages of disease development. We randomised across a variety of factors such as geographic location, disease stage, crop variety, and air temperature. The two datasets were collected with and without a 720‐nm neutral density filter to block all visible light. For the RGB images, the factory camera settings were used with the “intelligent‐auto” program. Infrared images were collected with a longer exposure time, and the following camera settings were applied—ISO: 200; white balance: 2000 kelvin; noise reduction: On; noise filter: low; shutter speed: auto; delay: 12 seconds. These images were collected at two research stations, on either side of the Andes. The research stations, belonging to Instituto Nacional de Investigaciones Agropecuarias (INIAP), were located at Pichilingue and Coca, Ecuador.

### Model development and optimisation

**Figure 1:**
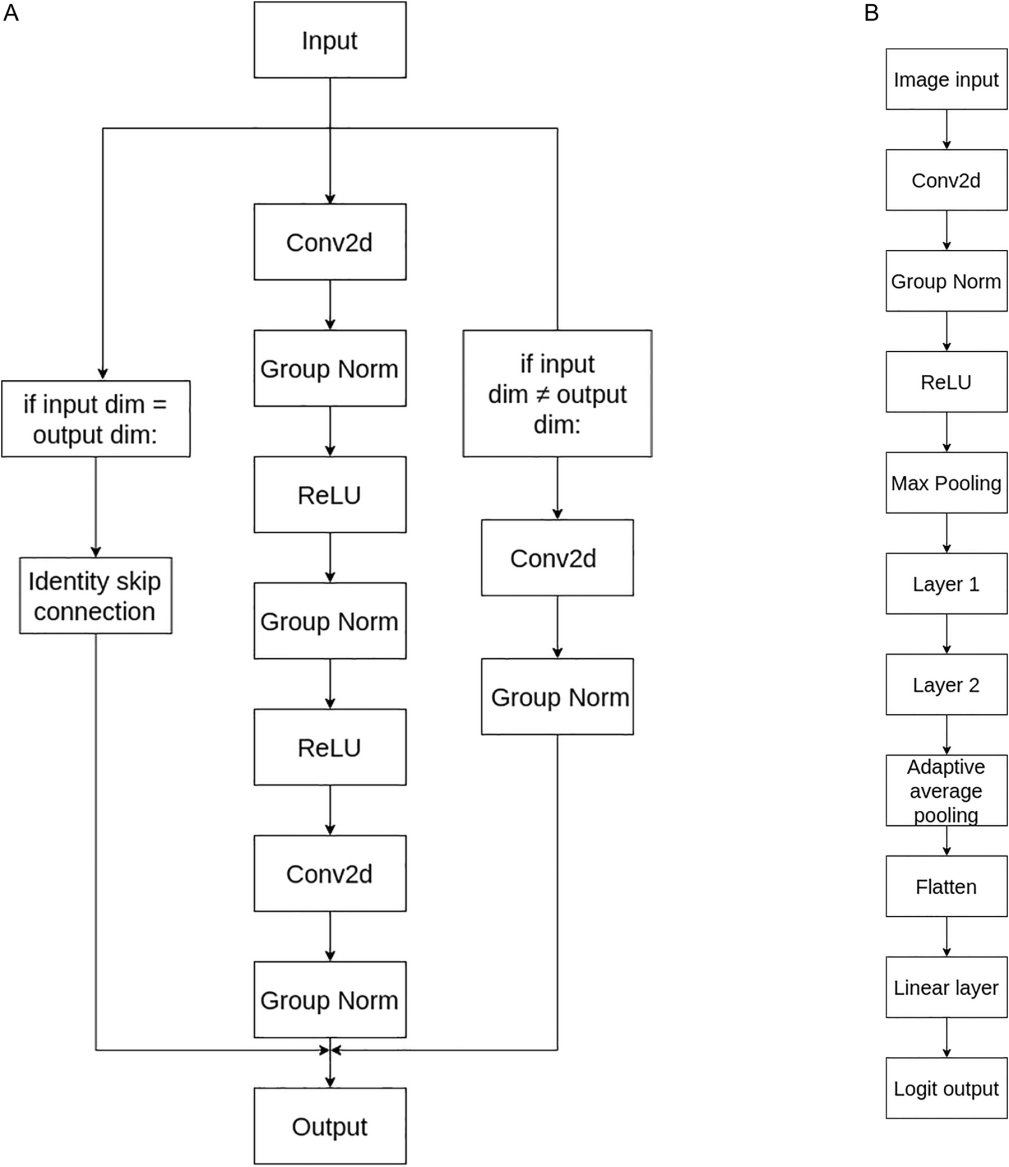
Schematic diagram of the PhytNet convolutional neural network. (A) PhytNet convolutional block design incorporated into PhytNet as sequential blocks in layers 1 and 2. (B) Full PhytNet end‐to‐end design. Layer 1 and layer 2 are composed of a predefined number of sequential convolutional blocks. The number of channels and kernel size of all convolutional layers should be optimised for a given dataset, but these optimised values are constant within layer 1 and layer 2, respectively. The stride values of layer 1 and layer 2 are 1 and 2, respectively. ReLU, rectified linear unit.

In PhytNet, various parameters, described below, are determined by a configuration file. This allows the model configuration to be easily optimised for a given dataset using a guided optimisation sweep. Similar to EfficientNet, PhytNet concludes with an adaptive average pooling layer before a fully connected layer, which allows for easy scaling and variable image input size post‐training. The PhytNet architecture, illustrated in Figure 1, is designed to be easily configured for specific datasets, with design choices that reflect a balance between capture of fine‐grain detail, avoidance of overfitting, accuracy, and computational efficiency. The convolutional block of PhytNet is shown in Figure 1A; an optimised number of these blocks are applied sequentially in layer 1 and layer 2 of PhytNet (Figure 1B). The number of channels and kernel size of all convolutional layers are also optimised for a given dataset, but these optimised values are constant within layers 1 and 2, respectively. The stride values of layer 1 and 2 are 1 and 2, respectively. This forces the earlier layer block(s) to focus on more fine‐grained details of the image than layer 2 and reduces the computation cost of layer 2. Figure 1A also shows two options for the skip connection. The standard identity skip connection on the left is used if the input dimensions of the block are the same as the output dimensions. However, in order to allow for flexibility in kernel size and convolutional dimensions in the optimisation sweep, the convolutional skip connection may adjust the dimensions of the input to match the output without applying an activation function. The convolutional block also automatically pads the skip connection output if necessary to allow for this process. While these two “if” statements add computational cost, they allow for great flexibility in model scaling.

PhytNet uses group normalisation in place of batch normalisation. Unlike batch normalisation, which normalises across the batch dimension, group normalisation divides the channels into groups and normalises within each group. This can be particularly useful for smaller batch sizes and is a deviation from the standard ResNet design. Additionally, batch normalisation has been shown to be suboptimal for plant pathology (Gong et al., 2021; Sykes et al., 2024). Except for one max pooling layer, pooling is avoided in PhytNet to preserve fine‐grained detail in the image, which is vital to disease detection.

Starting with a basic residual CNN and the intentionally small dataset of 240 infrared images, a series of training runs were executed while various configurations were manually tuned and tracked using the Weights & Biases platform (wandb; San Francisco, California, USA; https://wandb.ai/). These configurations included different activation functions such as rectified linear units (ReLUs) and Gaussian error linear units (GELUs) (Hendrycks and Gimpel, 2016), attention mechanisms including multi‐head attention (Vaswani et al., 2017) and squeeze‐excitation layers (Hu et al., 2019); dimension reduction such as max pooling, average pooling, and adaptive average pooling; convolution block/bottleneck block configurations; stochastic depth (Huang et al., 2016); dropout (Hinton et al., 2012); batch normalisation (Ioffe and Szegedy, 2015); layer normalisation (Ba et al., 2016); group normalisation (Wu and He, 2018); model depths; and dense layer configurations. Any adjustments to the architecture were retained if they improved the validation F1 score while reducing, or at least maintaining, signs of overfitting. Table 1 shows the results of a post hoc ablation analysis in which we replaced each of the model features shown, such as model layers or activation function, with alternatives. GELU is now commonly used in advanced architectures such as ViT, ConvNeXt, and GPT‐2 because it provides a smoother function than ReLU and does not suffer from non‐differentiability at zero; however, it is more computationally expensive than ReLU (Hendrycks and Gimpel, 2016; Liu et al., 2022). The authors of ConvNeXt chose to use GELU despite finding that substituting ReLU for GELU did not affect accuracy, while we found it had a deleterious effect on F1 score (Table 1). For these reasons, we used ReLU in PhytNet.

**Table 1:** Results of an ablation analysis comparing the results of substituting model features relative to the baseline PhytNet model shown in Figure 1.

Model	Training F1	Validation F1
Baseline	0.724	0.679
ReLU → GELU	0.390	0.378
Max pool → Average pool	0.348	0.313
Baseline + SE layer	0.357	0.369
Group normalization → Batch normalization	0.683	0.455
Group normalization → Layer normalization	0.771	0.640

*Note*: GELU = Gaussian error linear unit; ReLU = rectified linear unit; SE = squeeze excitation.

In addition to performance metrics such as accuracy, F1 score, and loss, the number of trainable parameters and giga floating point operations per second (GFLOPS) were also recorded for each model. Choosing a model with a high valuation F1 score, comparatively low training F1 score, and the minimum number of trainable parameters and GFLOPS would help to avoid overfitting and reduce computational cost. This strategy of model choice was also applied in the subsequent optimisation sweep. Once the base architecture was chosen, an optimisation sweep was run using wandb's Bayesian optimisation method to optimise for the validation set F1 score. Optimising for validation F1 score in this way should act to reduce overfitting as we searched for a model that performed best on the validation set, not the training set. The Bayesian optimisation strategy used here is a sequential process that is particularly useful for optimisation of the hyperparameters of models that are expensive to train, such as neural networks. In this case, Bayesian optimisation uses a Gaussian process to model the validation F1 score as a function of model hyperparameters. This sweep optimised the kernel size of the middle convolutional layer of each bottleneck block (1:19), number of convolution layer channels (16:128), number of bottleneck blocks in each convolution block (1:4), square image input size (200:500 pixels), learning rate (1–6:1–3), number of output channels (4:10), and beta1 (0.88:0.99) and beta2 (0.93:0.999) values of the AdamW optimiser (Loshchilov and Hutter, 2017), which control the exponential moving average of weight updating. These values were selected to facilitate the search for a model that would avoid overfitting, train in a controlled manner while allowing enough stochasticity to properly search the loss landscape, and have an appropriate kernel size to focus its attention on the features of the given dataset. The importance of kernel size optimisation is exemplified in ConvNeXt (Liu et al., 2022). During the sweep, models larger than 6 GFLOPS or two million trainable parameters were terminated before training. A total of 645 models were trained using one Nvidia GeForce GTX 1080 Ti GPU and one Nvidia Quadro P6000 GPU (Nvidia, Santa Clara, California, USA). Each model was trained on a single GPU, taking approximately 4 min to train. Early stopping was applied to halt training when the validation loss failed to decrease for 20 consecutive epochs, at which point the checkpoint with the best validation F1 was saved. Simple supervised training was used with AdamW optimisation (weight decay = 1^–4^ and eps = 1^–6^). L1 regularisation was added to the cross‐entropy loss function by summing the value of all model parameters and multiplying that value by a weight of 1^–5^. The following image augmentations were randomly applied during training: horizontal flip, Gaussian blur, and random rotation of 0–5 degrees. We also optimised for the number of output nodes and observed that the best‐performing models had seven or eight output nodes, despite having only four image classes. Potential explanations for this observation are given in the Discussion section.

### Model evaluation

To assess the performance of PhytNet, we compared it to four competing architectures: ResNet18, EfficientNet‐b0, EfficientNet‐V2s, and ConvNeXt Tiny. Each of these four architectures was trained using the procedure described above with the addition of an optimisation sweep for image input size, learning rate, and beta1 and beta2 values of AdamW. This allowed for a fair comparison between models, giving the competing architectures a chance to avoid overfitting and produce favourable results. To ensure reputability, torch backends were set to deterministic, torch random seeds were set to 42, and the data loader random seeds were set based on worker ID. Initial weights were generated using PyTorch default methods rather than using pre‐trained ImageNet weights to ensure a fair comparison between models as no ImageNet weights are yet available for PhytNet. Plant dataset–specific pre‐trained weights for PhytNet will be produced in a subsequent study. The performance metrics of the four models were estimated using 10‐fold cross‐validation, with the same data split for each model. While 10% of the data was reserved for validation during model development, the typical 10% test data was not used for evaluation for three reasons: (1) In this study, the images are highly consistent in their features, meaning that any subset would closely resemble the overall distribution of the training data, leading to a risk of pseudo‐replication; (2) This dataset is too small to justify discarding 10% of the training data for testing; and (3) While cross‐validation is more computationally expensive, it has been shown to be a more robust means of model testing. This is because it gives a more conservative estimate of model performance, while better reflecting real‐world conditions. Cross‐validation also allows for the assessment of the distributions of performance metrics, which can be used to detect overfitting (King et al., 2021). Finally, using Grad‐CAM (Selvaraju et al., 2017), class activation maps were produced to inspect the informative features used by each model. Grad‐CAM allows for the identification of areas of an image that are used by a CNN to inform decision‐making. It identifies the class of interest and computes the gradients of the class score with respect to feature maps produced from the final convolutional layer output. This information is expressed as a heatmap, which is superimposed onto the original image. Using this method allowed us to further assess the degree of overfitting in each model and catch naive behaviour that is not apparent in summary statistics.

## RESULTS

### Locating machine‐visible symptoms

**Figure 2:**
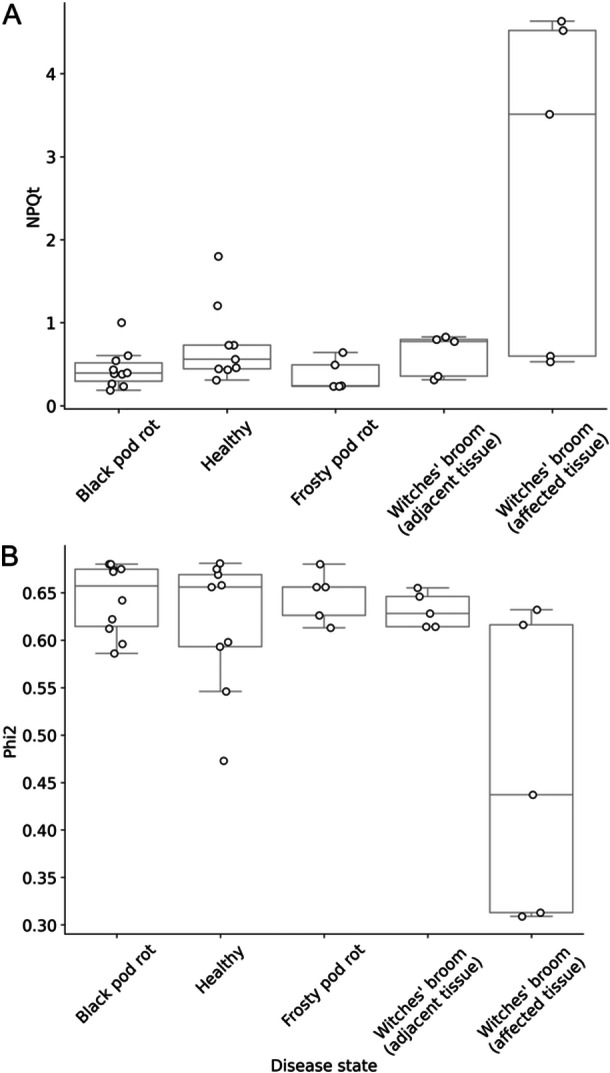
Distributions of non‐photochemical quenching (NPQt) (A) and photosynthetic yield (Phi2) (B) of cocoa trees with different disease states. Box plots show the interquartile range with whiskers at 1.5 times the interquartile range from the first and third quartiles. Raw data points are plotted as white circles. Measurements were taken from cocoa trees in five disease states using a MultispeQ v2.0. Black pod rot *n* = 10, Frosty pod rot *n* = 5, Healthy *n* = 9, Witches' broom (adjacent leaf) *n* = 5, Witches' broom (affected leaf) *n* = 5.

The results of measuring Phi2 and NPQt in diseased and healthy cocoa trees using a MultispeQ v2.0 are shown in Figure 2. These measurements were taken to assess if non‐visible signals of disease could be detected in the foliage of cocoa trees. While some of the leaves that were visibly infected with WBD showed clear but inconsistent signals of compromised photosynthesis, the leaves adjacent to pods and/or leaves infected with BPR, FPR, or WBD showed no signs of reduced Phi2 or increased NPQt relative to healthy trees. As such, we see no evidence that these diseases can be detected through compromised photosynthesis in foliage that shows no human‐visible signs of disease.

### Identifying informative light spectra

**Figure 3:**
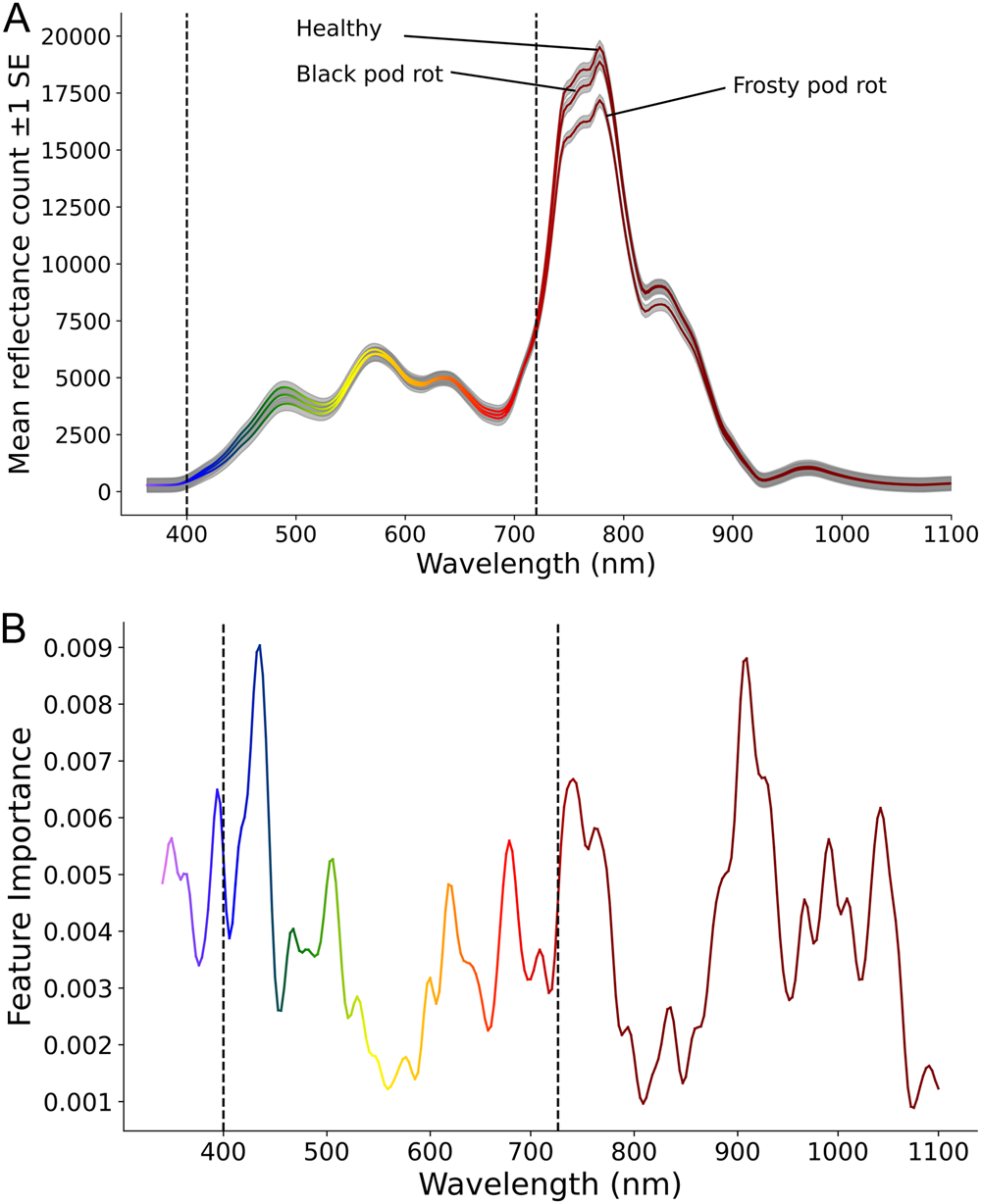
Mean reflectance spectrum measurements (±1 standard error) (A) and feature importance scores (B) derived from a random forest classifier trained on the same data. Spectroscopy data were gathered from cocoa pods that were either healthy or showing early to middle stage frosty pod rot or black pod rot symptoms. The standard error is shown by grey shading, and the dotted lines at 400 and 720 nm show the bounds of the human‐visible spectrum. Random forest classifier training accuracy = 95.45%; test accuracy = 78.26%.

The mean reflectance spectrum of cocoa pods and the relative importance of each spectrum in classifying disease are shown in Figure 3. While the healthy and BPR‐infected pods seem to show no difference in their reflectance spectrum (Figure 3A), the FPR‐infected pods show a clear reduction in infrared light reflectance. This may represent an informative signal to detect this disease, which can be asymptomatic until the late stages of development. While infrared light is among the most informative spectra (Figure 3B), the low importance scores shown here suggest that any single band of light spectra alone is not highly informative in classifying cocoa disease. However, the moderately high validation accuracy of the random forest classifier (78%) confirms that, taken together, spectroscopy data are informative. As shown in Figure 3B, many of the most informative light spectra are in the ultraviolet A (UVA; below 400 nm) and infrared range (above 720 nm), i.e., data that are mostly not captured in RGB images (Linhares et al., 2020). Additionally, the FPR per class accuracy (80%) from the random forest shows that the dip in infrared light of FPR is not sufficient alone to perfectly classify FPR, although the spike in feature importance between 700–800 nm suggests that it is informative.

### Model evaluation

**Figure 4:**
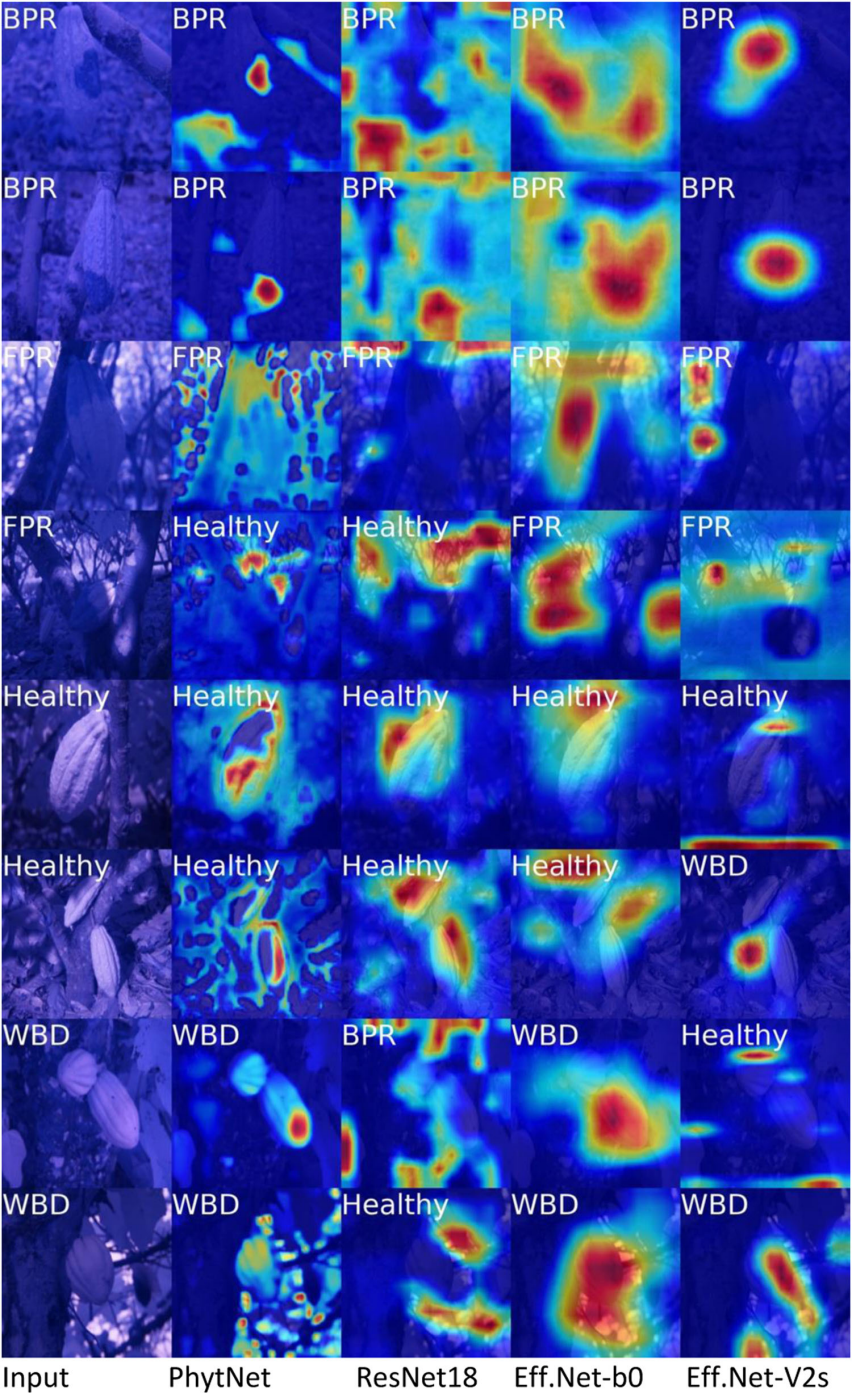
Infrared images with class activation heatmaps produced using Grad‐CAM and four convolutional neural networks. The models used are PhytNet, ResNet18, EfficientNet‐b0, and EfficientNet‐V2s (left to right). The leftmost column shows raw input images with ground truth labels in white; other white labels are predicted by each model.

While EfficientNet‐b0 and EfficientNet‐V2s give a relatively high mean validation F1 score: 69% (95% CI: 0.64–0.74) and 71% (95% CI: 0.66–0.75), respectively, both have a mean training F1 score 18 percentage points higher than their mean validation F1 score (Table 2), suggesting that they overfit to this data. This is corroborated by their higher validation loss than training loss and by the class activation maps produced using Grad‐CAM (Figure 4). Figure 4 shows that EfficientNet‐b0 focuses its attention poorly, except perhaps for the first WBD image. EfficientNet‐V2s seems to focus its attention quite well on the BPR images, although in both cases it seems to focus more on the healthy tissue than the disease lesions. Furthermore, EfficientNet‐V2s seems to fail completely to focus its attention on disease symptoms, or even the focal tree, in the other images, despite getting most classifications correct. This is a textbook definition of overfitting. That is, these models use image features that are consistent in the training set but are unrelated to the actual disease symptoms, potentially leading to poor generalisation to new or unseen data. Additionally, at 13.23 GFLOPS, EfficientNet‐V2s is far more computationally expensive than the other models described here, making it inappropriate for rapid classification when used on edge devices. ConvNeXt Tiny performed with poor F1 scores on this dataset (Table 2), although this is not surprising given its huge number of parameters (28.6 million) and the small size of this dataset. However, ConvNeXt Tiny's loss values were similar to those of ResNet18, and with an optimised input size of 212 pixels, its computational cost was measured at a very low 3.77 GFLOPS. Architectures like ConvNeXt and EfficientNet have been designed with great care to maximise computational efficiency. However, it is not high computational cost that leads to overfitting. High computational cost and model complexity are both, in part, functions of the number of parameters in a model. Despite being very computationally efficient, ConvNeXt Tiny remains a large model. It is this model size and complexity that, when paired with a small dataset such as ours, will result in overfitting. Considering the low F1 scores, it is clear that ConvNeXt is too large for the present dataset and so it is not considered in subsequent analyses here.

**Table 2:** Results of 10‐fold cross‐validation analysis comparing PhytNet to four competing architectures, all trained on the same infrared cocoa disease dataset.

Model	Training F1	Validation F1	Training loss	Validation loss	GFLOPS^a^	*n* pixels^2^	*n* parameters
PhytNet	0.60	0.61	0.93	1.17	1.19	285	336,196
ResNet18	0.62	0.65	1.8	1.94	6.16	408	11,178,564
EfficientNet‐b0	0.87	0.69	2.31	3.16	1.45	424	3,970,656
EfficientNet‐V2s	0.89	0.71	2.26	3.32	13.23	485	19,913,468
ConvNeXt Tiny	0.41	0.52	1.82	1.88	3.77	212	27,813,508

*Note*: GFLOPS = giga floating point operations per second.

^a^ GFLOPS were calculated using the optimised input size, shown here as pixels^2^. For this reason, GFLOP values reported by the original model authors may differ.

**Figure 5:**
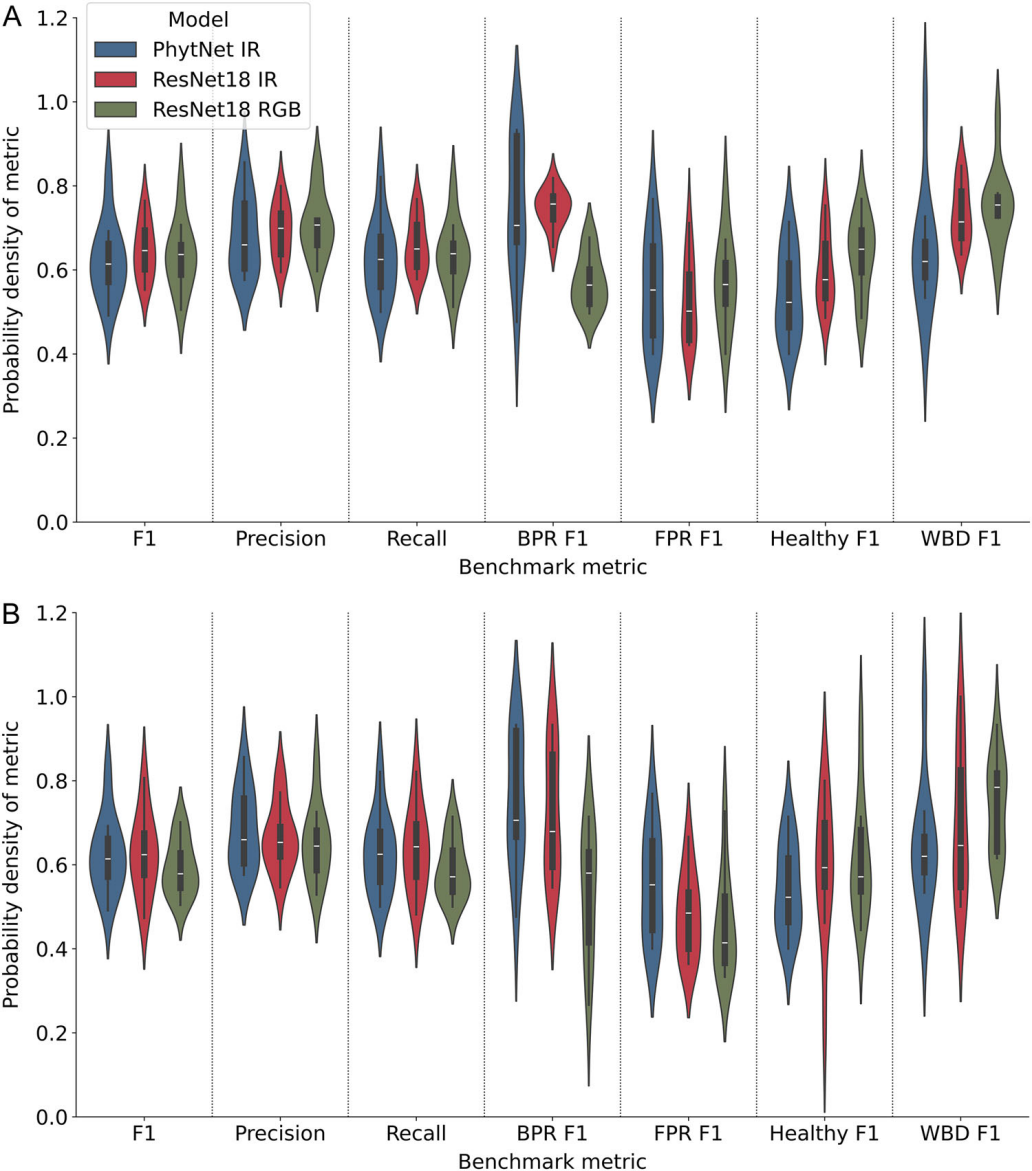
Violin and box plots of 10‐fold cross‐validation results for PhytNet and ResNet18 trained (A) and validated (B) on infrared or RGB images of cocoa disease. Shown here is the Gaussian density function, median and interquartile ranges for mean F1, per class F1, precision, and recall. PhytNet was trained only on infrared data, while ResNet18 was trained on infrared or RGB data. The datasets had four classes: Black pod rot (BPR), Frosty pod rot (FPR), Healthy, and Witches' broom disease (WBD). *n* = 70 images per class of early to midstage diseased or healthy cocoa with a 90%:10% train:validation split.

PhytNet and ResNet18 had almost perfectly consistent mean training and validation F1 scores, with PhytNet providing the lowest mean training and validation loss values. However, the difference between the training and validation loss values of PhytNet was the third highest in this comparison, which is an indication of slight overfitting. Additionally, as we will now discuss, these simple metrics are hiding naive behaviour on the part of ResNet18. As shown in Figure 4, ResNet18 focuses its attention slightly better than EfficientNet, while PhytNet focuses its attention on disease symptoms exceptionally well. In both FPR images in Figure 4, we also see that all four models seem to show some signs of being “distracted” or “overwhelmed” by the bright sunlight in the images, despite all four models making the correct classification in the first FPR image. However, despite this apparent distraction, PhytNet still shows that it focuses its attention on the cocoa pod or disease lesion. Figure 5 shows, with greater detail than Table 2, the results from the cross‐validation analysis of PhytNet trained on infrared images and ResNet18 trained on infrared or RGB images.

These two model architectures are compared here as they fit best to the data according to the summary statistics in Table 2 and the activation maps in Figure 4. ResNet18 was chosen to compare training on the infrared and RGB datasets as it is the better‐known and tested architecture. The median values per metric are very similar between models, with the exception of PhytNet's slightly higher validation F1 score for FPR (Figure 5). ResNet18 seems to have a higher median validation F1 score for the “Healthy” class (but with very long tails) and for WBD, meaning that its performance on the validation set was highly variable for these classes. Additionally, we see that the distribution of ResNet18 F1 values across classes for the training set is much narrower than in the validation set, with an almost perfect Gaussian distribution for the training F1 values for BPR. This indicates some degree of overfitting, where ResNet18 is learning the training data very well but struggles to generalise consistently across the validation set. Comparing the PhytNet training and validation results, the relatively Gaussian shapes of the distributions for F1 values, precision, and recall suggest that PhytNet is providing consistent and reliable results across the dataset. This consistency is further reinforced by the similarity between training and validation distributions. The wide tails in the PhytNet per class F1 scores are a concern as they indicate that its performance for specific classes is quite variable. However, considering the very small size of the dataset used here (per class training: *n* = 63, validation: *n* = 7), this variability in model performance is likely due to inconsistencies and noise in the data for specific classes, affecting the model's performance. The identical distributions between the training and validation set for PhytNet suggest that the model is not memorising the training data but is instead learning genuine patterns that apply to unseen data. Additionally, the ResNet18 mean training F1 score for FPR was 13% lower than the mean FPR validation F1 score. This curious behaviour, in conjunction with the failure of ResNet18 to consistently isolate features of interest in the Grad‐CAM analysis and the inconsistent distributions of values between the training and validation sets, suggests that ResNet18 is overfitting, if to a lesser extent than the EfficientNet variants. Figure 5 also shows a slight improvement in FPR classification using the infrared images vs. the RGB images. This, in conjunction with the 80% FPR accuracy of the random forest, corroborates the importance of the dip in infrared reflectance of FPR pods seen in Figure 3A. However, it should be noted that the accuracies of the BPR and Healthy classes were equally high with the random forest, despite no such obvious signal in the reflectance spectrum. We also see in Figure 5 that the BPR F1 score distribution was markedly lower when using the RGB dataset, while the median WBD F1 score was much greater.

## DISCUSSION

### Photosynthetic activity as a disease indicator

We observed no significant changes in Phi2 or NPQt between healthy leaves and those adjacent to diseased pods or leaves. This suggests that, if there are systemic effects of these diseases on photosynthesis, they are not readily detectable, at least in the early stages. This result reinforces the need to focus on localised symptoms for disease detection in cocoa, which is consistent with what we know of the pathology of these diseases. That is, while *Phytophthora* spp. can cause seedling blight and trunk cankers in adult trees, BPR and FPR symptoms tend to be isolated to the infected pods (Krauss, 2012; CABI, 2021). However, the sample size of this experiment is limiting and so, although the effect sizes seem to demonstrate a clear pattern, these results should be considered accordingly.

### Spectral characteristics of cocoa pod diseases

The reduction in infrared light reflectance in FPR‐infected pods observed here could be an important finding. This is because FPR often remains asymptomatic during its biotrophic phase, which lasts until the later stages of disease progression (Bailey et al., 2018), making early detection exceedingly difficult. This reduced infrared reflectance may be indicative of altered metabolic activity within a pod resulting from FPR infection. This is in contrast to BPR, which primarily affects the external pod surface, leaving the internal metabolic activities relatively unaltered until the later stages of disease progression (Akrofi, 2015). The spectroscopy data proved to be substantially informative for disease classification, especially in the ultraviolet–blue and red–infrared range. The feature importance scores presented here highlight that the most informative spectra lie in the UVA–blue and red–infrared ranges, which are not typically captured in standard RGB images. This suggests that computer vision models relying solely on RGB images may face challenges in accurate disease classification and should incorporate additional spectral data or rely more on morphological characteristics. This result is somewhat corroborated by the observation that ResNet18 trained on infrared images had slightly better median per class BPR and FPR F1 validation scores than when trained on RGB data. While we randomised across factors such as disease stage and crop variety, a more controlled analysis should be undertaken to confirm these findings. A study that more carefully controlled for factors such as air temperature, internal pod temperature, and crop variety differences would be highly informative. An additional informative feature for disease detection via computer vision that should be considered in future studies is polarised light. Polarised light can indicate plant cuticle properties related to stress and disease (Nilsson, 1995; Gunasekaran, 1996). However, while polarised light has been used in conjunction with CNNs to successfully map rice paddies (Bem et al., 2021), little such work appears to have been done since the advent of CNNs for plant disease detection.

### Evaluation of convolutional neural networks

The EfficientNet variants yielded high F1 scores but also showed clear signs of overfitting. In contrast, PhytNet and ResNet18 demonstrated more consistent performance in F1 scores and Grad‐CAM analysis. PhytNet performed by far the best in the Grad‐CAM analysis, while also having the lowest loss values and least GFLOPS.

The effects of bright sunlight may partly explain why the median FPR F1 score was relatively low in all models here. Because the causative agent of FPR, *M. perniciosa*, is wind dispersed (Leach et al., 2002), FPR tends to be found above 2 m in the canopy. This means that bright light will be more common in these images than in images of the soil‐borne BPR (Noble and Coventry, 2005) and may suggest a need for controlled imaging conditions. However, the fact that all four models correctly classified the first FPR image in Figure 4, despite this apparent distraction, suggests that the models might still leverage other features for classification when bright light is present. While PhytNet focuses on the diseased pods in addition to bright light, the other three models focus on irrelevant features such as the tree trunk, background, or only bright light. However, despite this, PhytNet classifies the second FPR image as healthy, possibly because only healthy pod tissue is brightly illuminated. While PhytNet had the lowest mean validation F1 score, it is the only model here that showed almost no signs of overfitting, it performed with great consistency between the training and validation sets, and it performed best for FPR images. This latter point is of high potential economic value to cocoa farmers. Furthermore, PhytNet focused its attention on relevant features with far greater specificity than the other models. This unique ability to focus exactly on the disease lesion is likely due to the optimisation of kernel size for the dataset and the low stride values in the convolutional blocks. Considering all of this, PhytNet would most likely perform best and most consistently in the field out of the models presented here.

### Optimisation of output node number

During the optimisation sweep, PhytNet performed consistently better with seven or eight output nodes, despite having only four classes in the dataset. In challenging classification problems, having more output nodes than classes offers several potential advantages. Extra nodes may capture nuanced feature representations (Donahue et al., 2017), act as “soft clusters” for variations within classes, or serve as a form of regularisation to improve generalisation through increased complexity of model parameters (DeVries and Taylor, 2017). The extra nodes could also provide a “catch‐all” for unknown or less frequent features, thereby preventing forced guesses and acting to prevent representation collapse, where disparate inputs are mapped to the same point or a very narrow region in the feature space (Goyal et al., 2020). Additionally, a larger parameter space may smooth the optimisation landscape, a characteristic that is said to make it easier for algorithms to find a good solution (Walshaw and Everett, 2002; Kell, 2012). These potential explanations for this curious model behaviour should be explored in future work.

## CONCLUSIONS

PhytNet, while promising, has potential limitations that have yet to be tested. In future studies, we will test if its performance is dependent on the dataset used and how well it captures intricate patterns in more complex data. Although efficient, its reduced complexity might be a compromising factor, and it could be prone to underfitting in certain scenarios. We will test PhytNet's ability in transfer learning in a future study, although with a larger plant pathology dataset rather than ImageNet, which is irrelevant in this context. Through the use of classical model evaluation techniques as well as class activation maps and cross‐validation, we show that PhytNet is a promising candidate model architecture, particularly in its ability to focus its attention exceptionally well on relevant features like cocoa pods and disease lesions. By using the qualitative analysis of Grad‐CAM in conjunction with quantitative analyses, we were able to successfully tease apart the behaviours of different model architectures in a way that clearly highlights their pros and cons. PhytNet is approximately five times less computationally expensive than ResNet18, and its superior attention, coupled with an apparent complete lack of overfitting, offers unique advantages that could be leveraged for specific applications, such as the localisation of disease symptoms on a tree. In automated fungicide application systems, the ability to accurately pinpoint the location of the disease symptom or pathogen could lead to more efficient and targeted application of fungicides, thereby reducing waste and pollution.

## AUTHOR CONTRIBUTIONS

J.R.S. conceived of this study, read and summarised the relevant literature, gathered the data, conducted the analyses, and wrote the first draft of the manuscript. K.J.D. and D.W.F. had substantial input in experimental and data analysis design, continually reviewed and edited the manuscript, and approved the final manuscript before submission and publication.

## Data Availability

All data described in the study can be freely accessed at: https://osf.io/sd7wf/?view_only=8dba47127e65454c98db9ea5bcf3501c. The code to optimise and train PhytNet for your data can be found at: https://Github.com/jrsykes/PhytNet.
